# Relationships among Student-Athletes’ Identity, Mental Health, and Social Support in Japanese Student-Athletes during the COVID-19 Pandemic †

**DOI:** 10.3390/ijerph18137032

**Published:** 2021-06-30

**Authors:** Goichi Hagiwara, Takaaki Tsunokawa, Takehiro Iwatsuki, Hironobu Shimozono, Tsuyoshi Kawazura

**Affiliations:** 1 Department of Human Science, Kyushu Sangyo University, 2-3-1 Matsukadai, Higasgi-ku, Fukuoka-shi, Fukuoka 813-8503, Japan; 2 Department of Human & Engineered Environmental Studies, Graduate School of Frontier Science, The University of Tokyo 5-1-5 Kashiwanoha, Kashiwa, Chiba 277-8561, Japan; 3 Department of Health and Sport Science, University of Tsukuba, 1-1-1 Tennodai, Tsukuba-shi, Ibaraki 305-8574, Japan; tsunokawa.takaaki.ke@u.tsukuba.ac.jp; 4 Department of Kinesiology, The Pennsylvania State University, Altoona College, 3000 Ivyside Park, Altoona, PA 16601, USA; tui36@psu.edu; 5 Department of Sports and Health Science, Fukuoka University, 8-19-1 Nanakuma, Jonan-ku, Fukuoka-shi, Fukuoka 814-0180, Japan; cmo@fukuoka-u.ac.jp; 6 Department of Sports Science, Kyushu Kyoritsu University, 1-8, Jiyugaoka, Yahatanishi-ku, Kitakyushu-shi, Fukuoka 807-8585, Japan; tsuyoshi@kyukyo-u.ac.jp

**Keywords:** athletic program, collegiate sports, UNIVAS, COVID-19, mental management

## Abstract

The purpose of the two studies was to investigate the relationships among student athletes’ identity and mental health during the COVID-19 pandemic. In addition, this study aimed to clarify the relationship between perceived social support from teammates and mental health in student-athletes. Two studies were conducted to investigate and clarify the mental health states of student-athletes in Japan during the COVID-19 pandemic. In Study 1, conducted in April 2020, the participants were 402 male student-athletes and we examined the relationships among student-athletes’ identity and mental health. The results of correlational analyses indicated significant negative correlations between the degree of student-athletes’ identity and depression and sports helplessness. In Study 2, conducted in March 2021, the participants were 135 male student-athletes and examined the relationship between perceived social support from teammates, student-athletes’ identity, and mental health. The results indicated a significant correlation between social support, student athletes’ identity, and mental health.

## 1. Introduction

The Japan Association for University Athletics and Sports (UNIVAS)—the national governing body for intercollegiate sports—was established in 2019. The UNIVAS aims to ensure the safety of student-athletes and has started offering programs to support the physical and mental health of student-athletes in Japan. However, in April 2020, shortly after the UNIVAS started its support program, the COVID-19 pandemic caused most Japanese universities to close their campuses and move their classes online, leaving many student athletes unable to play sports or study satisfactorily. Many student-athletes were then unable to participate in their sports. Therefore, there is an important need to study mental health during the COVID-19 pandemic and to better understand the psychological and emotional perspectives among student-athletes in Japan.

Athletic identity has been defined as “the degree to which an individual identifies with the athletic role” [1]. Athletic identity is often used to examine the social identity of student-athletes and encompasses the psychological, emotional, and behavioral components of an athlete’s self-identity [2]. The relationship between athletic identity and mental health has been examined. Miller and Hoffman [3] found that athletic identity was negatively related to symptoms of depression. Furthermore, Graupensperger et al. [4] stated that it is also important to consider the importance of athletic identity when investigating the impact on mental health among student-athletes during the COVID-19 pandemic in the United States, and also found a negative correlation between athletic identity and depressive symptoms. The higher degree of athletic identity tended to lead to better mental health states [4,5,6]. However, the relationship between student-athletes’ athletic identity, and mental health under the COVID-19 pandemic has not been examined in Japan. Therefore, two studies are conducted to better understand the relationship between athletic identity and mental health and to examine the factors that positively influence this relationship.

The purpose of Study 1 was to investigate the relationships between athlete’s identity and mental health in Japanese intercollegiate student-athletes during the first COVID-19 pandemic (April 2020) in Japan. In addition, this study examined the degree of identity of Japanese student-athletes based on their year in college, as athletic identity can be investigated from a developmental perspective [1]. Several previous studies have examined the degree of athletic identity in a time series [7,8,9]. In the study of student-athletes, the first-year students had a higher degree of athletic identity compared with fourth-year students [8]. In addition, in a study of former and current student-athletes, athletic identity decreased during the fourth year of competition as they neared retirement [7]. On the other hand, athletic identity increased at the peak of athletic performance [10,11,12]. In Japan, due to the start of the new school year in April, and the fourth year of college is the start of the final year of school. Therefore, we assumed that student-athletes’ identity would be especially high in the fourth year of college.

This study would also examine the relationship between different degrees of athletic identity and mental health based on year in universities. Graupensperger et al. [4] noted that it is important to evaluate athletic identity under the COVID-19 pandemic to assess its impact on student-athletes’ mental health. They showed that social support with teammates might be related to mental health and athletic identity. Lakey and Cohen [13] also emphasized that several perspectives on “social support” locate identity as a mechanism linking supportive relations with significant others and mental health. Therefore, it can be inferred that there is a relationship between social identity, mental health, and social support with significant others. It would be possible to approach the issue of student athletes’ mental health by examining these three factors.

Social support has gained attention from researchers as a beneficial resource for particular health-related issues [14]. Generally, social support is defined as “the help provided by individuals who comprise the social network of a person who occupies a position of ego in this network” [15]. According to Hisada [16], the major findings on social support show that “various forms of support provided by others should play the major role in maintaining or improving the person’s mental health”. Drawing on these findings, many researchers have begun focusing on the relationship between receipt of social sup-port and mental health [17,18,19,20,21]. This line of research has produced a number of important findings. For example, social support has been shown to reduce stress, anxiety, and helplessness related to engaging in, or continuing, competitive sports [22,23]. Thus, social support from significant others might be beneficial for maintaining the mental health of intercollegiate student-athletes.

In addition, Erikson [24], a pioneer in identity research, stated that in the process of identity formation, especially in adolescence, the personality is formed by clarifying the position of the self in the social context of the nation, culture, and relationships with significant others. It can be inferred that the process of individual identity formation differs depending on social factors. In addition, relationships with significant others in social contexts such as family, peers, school, and work are deeply related to the formation of adolescent identities [25]. The relationships that adolescents have with their significant others, such as family members and peers who live in the same context as themselves, affect their social identity formation. From this standpoint, the field of social psychology has been examining the relationship between identity formation and social support. Caldwell et al. [26] examined the degree of identity and satisfaction with the recognition of social support from significant others and found that the higher the degree of recognition of social support indicated the higher the degree of identity. In Japan, the higher the level of recognition of social support demonstrated the higher the level of identity in college students [27]. Thus, social support from others may have an impact on the formation of identity, and research focusing on social support has been increased.

In the field of sport science, Hagiwara and Isogai [28] examined the relationship between the degree of perceived social support relationship with teammates and athletic identity among student-athletes. The relationship between perceived social support from team-mates and athletic identity was found to be higher for student-athletes who perceived receiving social support from teammates. As described above, college sport research has shown that social supports from teammates on sport teams are related to formation of identity as a student-athlete.

In Japan, the first state of COVID-19 emergency was declared in April 2020, forcing student athletes to suspend their activities, practices, and competition. In addition, a second state of COVID-19 emergency was declared in January 2021, forcing the cancellation of campus and athletic activities. And as of the end of February 2021, the state of the COVID-19 emergency has been lifted and activities and practices at the university have begun to resume. With the resumption of athletic club activities at the university, student athletes are gradually freeing themselves from social isolation as they resume interaction with their teammates. Therefore, the purpose of Study 2 was to add social support as it relates to mental health and aimed to better understand the relationship among social support from teammates, student-athletes’ identity, and mental health. In addition, we compared student-athletes’ identity and mental health status from surveys conducted at the time of the first and second COVID-19 emergency declarations.

## 2. Method

### 2.1. Participants

In Study 1, we conducted a web-based survey at five different universities (five teams of track and field, swimming, and judo, respectively) that have top-level collegiate athletic clubs in Japan and provide scholarships to their student-athletes. The web-based survey was only conducted for those teams that had obtained prior consent from the team managers. Participants were 402 male Japanese collegiate student-athletes (Mean age = 19.72, SD = 1.37). Their sporting activities included track and field (192), swimming (113), and judo (97). One-hundred and five first-year, 109 second-year, 83 third-year, and 105 fourth-year university student-athletes were recruited. They belonged to the collegiate athletic team and had participated in national collegiate championships. The national-level participants were those who had participated in national collegiate championships while attending university. In addition, they received scholarships from universities to enable them to balance between their academic and athletic activities.

In Study 2, we had planned to conduct a survey with the same five university subjects as in Study 1. However, all teams had not resumed their club activities as of March 2021. Therefore, we conducted a web-based survey at three universities (three basketball teams and two rugby football teams) where we newly requested the survey and obtained the consent of the team managers. These universities also had national-level athletic clubs and offered scholarships to their student-athletes. Participants were 135 male Japanese collegiate student-athletes (mean age = 20.01, SD = 1.11). Only student-athletes who had resumed their club activities practiced and played games were included in this study. Their sporting activities included basketball (99) and rugby football (36), and 50 were first-year university students, 41 were second-year, and 44 were third-year. The fourth-year of university students were not included in the survey because they had already retired from athletic club activities. The participants recruited for Study 2 were also scholarship student-athletes with the same conditions as in Study 1.

### 2.2. Instruments

Athletic identity is often used to examine the social identity of student-athletes. Brewer et al. [1] originally developed the Athletic Identity Measurement Scale (AIMS), and several studies have conducted cross-cultural psychometric evaluations of the AIMS [10,11,29,30,31]. Thus, the AIMS is the most widely used measure of athletic identity worldwide [29]. In Japan, Hagiwara and Isogai [9] developed a Japanese version of the AIMS to demonstrate the extent of athletic identity in Japanese student-athletes. Athletic identity is an important component to better understand athletes from a psychological and emotional perspective. However, while the AIMS [1] measures the degree of social identity as an athlete, it does not measure the degree of identity as a student-athlete with the dual roles of academics and athletics [6,12,32]. Harrison et al. [33] constructed a new measure called the Baller Identity Measurement Scale (BIMS), an adaptation of the AIMS and indicated it as a measure of the student-athlete’s identity in the United States. Similar to the AIMS, several studies have also conducted cross-cultural psychometric assessments of the BIMS to assess the degree of student-athlete identity [12,32,33,34]. Therefore, in Study 1 and Study 2, the Japanese version of the BIMS (BIMS-J) [32] was used to measure the degree of student-athletes’ identity. This questionnaire consisted of 10 statements (e.g., “I consider myself a student-athlete”; “Other people see me mainly as a student-athlete”) that were rated on a 7-point Likert scale with response options ranging from 1 (strongly disagree) to 7 (strongly agree). Support for the reliability and validity of the measurement were confirmed via Cronbach’s alpha and confirmatory factor analysis [32].

In Study 1 and Study 2, the depression and sports helplessness subscales of the Stress Response Scale for Athletes [35] were used to measure the athletes’ mental health issues. Each subscale begins with the following sentence: “To what extent have you experienced the following within the past two to three weeks?” The question is then followed by items such as “I feel depressed” or “I cannot find a meaning in the purpose of playing sports.” Each item was rated on a five-point scale ranging from 1 (completely disagree) to 5 (completely agree). The reliability and validity of the measurement were confirmed via Cronbach’s alpha and confirmatory factor analysis [35].

In Study 2, the Receiving Social Support Scales for Sports Teams [36] questionnaire was used to measure perceived social supports from teammates. This questionnaire assesses individuals’ perceptions of receiving social support from their teammates. The scale consists of six items, which are preceded by the sentence, “We would like to ask you about the help and support you receive from your teammates when you play or decide to continue playing competitive sports. Your teammates…” Example items include “give you advice to help solve your problems” and “cheer you up when you are feeling low.” These items are rated on a scale of 1 (strongly disagree) to 5 (strongly agree) and are summed to provide a total score representing the amount of received social support. For this social support scales, evidence of reliability and validity of the measurement were confirmed via Cronbach’s alpha and confirmatory factor analysis [36].

The reliability and validity of all scales used in this study were based on Cronbach’s alpha (>0.70) and goodness-of-fit indices (GFI, AGFI, and CFI < 0.90 and RMSEA < 0.08).

### 2.3. Procedure

The institutional review board approved this study of the National Institute of Fitness and Sports in Kanoya (No.8-1, April 2020). All participants were informed of the instructions and the purpose of this study during an online meeting of their athletic activity, and their participation was voluntary. We contacted the club manager of each university in advance and conducted a web-based survey on only those student-athletes who had received scholarships from their university.

In Study 1, a web based-survey was conducted in early April 2020, a time when academic and athletic activities at the university were suspended due to the COVID-19 pandemic. In Study 2, a web-based survey occurred in early March 2021. During this period, the declaration of the state of emergency due to the second COVID-19 pandemic was lifted, and some universities were able to conduct athletic club activities and other activities.

### 2.4. Data Analysis

In Study 1, descriptive statistics were calculated for each measure. One-way ANOVA analyses were used to compare student-athletes’ identity, depression, and sports helplessness by year in the university. Pearson’s correlation coefficients were used to examine the relationships among the degree of student-athletes’ identity, depression, and sports helplessness by year in the university. In Study 2, one-way ANOVA analyses were used to compare among student-athletes’ identity, depression, sports helplessness, and perceived social supports from teammates by year in the university. In addition, Pearson’s correlation coefficients were used to examine the relationships among the degree of student-athletes’ identity, depression, and sports helplessness perceived social supports with teammates by year in the university. Finally, we conducted a t-test to compare the student-athlete’s identity and mental health status at the first and second time of the COVID-19 emergency declaration using the data from Study 1 and 2. All data were analyzed using IBM SPSS Statistics 27.0.

## 3. Results

### 3.1. Descriptive Statistics

In Study 1, the descriptive statistics of student-athletes’ identity, depression, and sports helplessness are shown in Table 1. Results of the one-way ANOVA analysis showed that there were significant differences in sports helplessness between each university year (*F*(3, 398) = 5.32, *p* < 0.001, *η*^2^ = 0.14). In addition, in the results of Tukey post-hoc test, there were significant differences between first-year, second-year, and fourth-year student-athletes.

In Study 2, the descriptive statistics for student-athletes’ identity, depression, and sports helplessness are shown in Table 2. Results of the one-way ANOVA analysis showed that there were no significant difference in student-athletes’ identity, depression, and sports helplessness.

### 3.2. Relationships between Student-Athletes’ Identity, Depression, and Sports Helplessness

In Study 1, correlational analyses indicated that there were significant negative correlations between student-athletes’ identity and depression among third-year and fourth-year student-athletes. During the second-year, third-year, and fourth-year, student-athletes’ identity had a significant correlation with sports helplessness. In addition, there were significant correlations between depression and sports helplessness (Table 3).

### 3.3. Relationships between Received Social Support from Teammates, Degree of Student-Athletes’ Identity and Depression, and Sports Helplessness

In Study 2, correlational analyses indicated, that there were significant negative correlations between student-athletes’ identity and sports helplessness in all years of student-athletes. In addition, there were positive significant correlations between student-athletes’ identity and received social supports from teammates in all years of college. On the other hand, there was no significant correlation between student-athletes’ identity and depression in all years of college. In the relationship between social support and depression, a significant negative correlation was found among first-year, but no significant difference was found among second and third-year students. In the relationship between social support and sports helplessness, there were significant negative correlations among first and third-year students; on the other hand, there was no significant difference among second-year students. In addition, there were significant correlations between depression and sports helplessness (Table 4).

### 3.4. A Comparison of Student-Athlete Identity and Mental Health Status from Surveys Conducted at the First and Second Time of the COVID-19 Emergency Declaration

A comparison of student-athletes’ identity and mental health status from surveys conducted at the first and second time of the COVID-19 emergency declaration showed that although there was no significant difference in sports helplessness (t = 0.38, df = 535, *p* = n.s.), student-athletes’ identity (t = 4.09, df = 535, *p* < 0.001) and depression (t = 2.98, df = 535, *p* < 0.01) were significantly higher in the second survey subjects than in the first (Table 5).

## 4. Discussion

The purpose of these two studies was to better understand the relationship between athletic identity and mental health and examine the factors that influenced these during the COVID-19 pandemic in Japan. Specifically, Study 1 examined the relationships among identity, depression, and sports helplessness. Study 2 examined the social support that positively affects identity and mental health one year after the first COVID-19 pandemic in Japan. In addition, this study compared student-athlete identity and mental health status from surveys conducted at the time of the first and second COVID-19 emergency declarations.

Both Study 1 and 2 indicated no significant difference in student-athletes’ identity by college year. Previous studies have shown that athletic identity increased during peak athletic performance and declined during retirement from competition [8,37,38,39]. Since performance usually peaks for the last college year in Japan, we expected that identity in fourth-year college student-athletes would be higher than for other years. However, this study did not show the tendency. This result is thought to be due to the cessation of athletic activities in university caused by the COVID-19 pandemic. Student-athletes in their fourth year were in the final year of their academic and athletic careers. They are at the termination of their athletic careers, but the COVID-19 pandemic has prevented them from playing satisfactorily. As a result, when the athletic identity is supposed to be strongly formed in the final year. This might indicate the same degree of identity as other years. In Study 1, there was no significant difference in depression; however, a significant difference was found in sports helplessness. The fourth-year students showed higher sport helplessness than the first- and second-year students. In Study 2, there was no significant difference in depression and sports helplessness by college year. Based on our findings, the risk of depressive symptoms increased at the time of athletic retirement, and this is in line with a previous study on the topic [40] and indicated that depression symptoms have emerged as one of the most common emotional reactions when an athlete is injured [41]. From this knowledge, we expected that athletes in the fourth-year athletes who were close to retirement from collegiate sports or athletes who could not play sports by the COVID-19 pandemic would have higher depressive tendencies than athletes in the other years; however, no difference was found. The fourth-year college students tended to be significantly higher in sports helplessness, which may be due to the impact of the cancellation of competitions and practices by the COVID-19 pandemic. Especially in the last year of intercollegiate sports, the COVID-19 pandemic was thought to dramatically increase this tendency.

Regarding the relationships among the degree of student-athletes’ identity, depression and sports helplessness by college year, there were significant correlations among these factors in Study 1 and 2. First, there were significant positive correlations between depression and sports helplessness in both studies. Previous studies have shown a relationship between helplessness and depression [42,43]. A relationship between the two factors was examined in cancer patients, and found that helplessness was a predictor for depression, and that feelings of helplessness might induce depressive symptoms [43]. Helplessness may be an antecedent of depressive feelings, and it was possible that the same might occur in athletes in this study. In particular, the fourth-year student-athletes experienced a high level of sports helplessness because of a decrease in motivation to continue competing by the closure of the university and the cessation of athletic activities due to the COVID-19 pandemic at the start of their final year of collegiate sports. Taken together, the COVID-19 pandemic may predict a mental health crisis in student-athletes.

Secondly, there was a significant negative correlation between student-athletes’ identity and mental health. In Study 1, a negative correlation was found between identity and depression in third- and fourth-year students. In addition, negative correlations were also found between identity and sports helplessness in above second-year students. In Study 2, there were significant negative correlations between student-athletes’ identity and sports helplessness in all students. On the other hand, there was no significant correlation between student-athletes’ identity and depression in all college years. The higher degrees of athletic identity tended to be associated with better mental health [4,6]. Graupensperger et al. [4] examined the relationship between mental health and athletic identity during the COVID-19 pandemic in American student-athletes and found that athletes who formed a strong athletic identity were associated with lower feelings of depression. This study indicated that student-athletes’ identity influenced the suppression of helplessness that is a predictor of depression in Japanese student-athletes. The results support the previous study among American populations suggest that the current situation of student-athletes around the world is similar. In addition, Miller and Hoffman [3] indicated that athletic identity is negatively associated with depression, and our study confirmed previous studies. These findings suggested the importance of maintaining the student-athlete identity—primary role or self-concept as a student and athlete—to maintain better mental health. Particularly, we found a moderate correlation between student-athletes’ identity and sports helplessness in the senior year. They were in their fourth year of collegiate sports, yet an unexpected interruption in competition due to the COVID-19 pandemic occurred. Some of them were forced to retire from college sports due to the COVID-19 pandemic, which might have increased their sports helplessness. The results also showed a strong correlation between depression and sports helplessness–associated with the helplessness that is an antecedent of depression in previous studies [42,43]. To prevent student-athletes from depression, supporting them to maintain their identity as student-athletes is imperative.

There were significant correlations regarding the relationships among social supports from teammates, student-athletes’ identity, and mental health. In the relationship between social support and depression, a significant negative correlation was found among the first year, but no significant difference was found among second-and third-year students. There were significant negative correlations among first- and third-year students in the relationship between social support and sports helplessness. On the other hand, there was no significant difference in second-year students. In addition, there were significant correlations between depression and sports helplessness. Moreover, there were positive significant correlations between student-athletes’ identity and received social support from teammates in all college years.

Previously, several studies mentioned a relationship between social supports, mental health, and social identity [4,13,21]. Hagiwara et al. [21] examined the relationships between social support and mental health among American collegiate student-athletes and indicated that received social support from teammates was negatively correlated with depression and sports helplessness. In addition, Graupensperer et al. [4] noted that it is important to consider the importance of athlete identity when examining the impact on student-athletes mental health during the COVID-19 pandemic. They demonstrated that social support with teammates might be related to mental health and athletic identity. Lakey and Cohen [13] also emphasized that several perspectives on “social support” locate identity as a mechanism linking supportive relations with significant others and mental health. As described above, previous studies inferred that there are relationships among social identity, mental health, and social support with significant others, and it can be inferred that this study followed these previous studies. Finally, comparing student-athletes’ identity and mental health status between the first and second times of the COVID-19 emergency declarations demonstrated that student-athletes’ identity and depression were significantly higher among participants in the second survey than those in the first survey. In Japan, the number of COVID-19 infections exploded in early April, August, and November 2020, and in January and April 2021, with more than 770,000 people infected as of June 2021 [44]. Japan has so far controlled the infection explosion by actively tracking clusters, limiting group gatherings, and advocating hand hygiene, but the number of infections has skyrocketed since November 2020, suggesting that there is no simple solution to this problem [45]. Even after the declaration of the state of emergency was lifted in late February 2021, few teams were able to hold proper club practices, and the mental state of the student-athletes was expected to be severely decreased. This situation might have resulted in a higher trend of depression among student-athletes in the March 2021 survey than in April 2020.

In addition, since all 402 and 135 participants in Study 1 and Study 2, respectively, were student-athletes in individual sports and team sports, we discussed differences between individual sports versus team sports. Costa et al. [11] compared the athletic identity of Italian athletes during the COVID-19 lockdown period between individual sports and team sports. They found that team sports’ athletes had a higher degree of identity than individual sports’ athletes. In addition, Uroh and Adewunmi [46] compared athletic identity and psychological distress between individual and team sports among Nigerian athletes during the COVID-19 lockdown. Individual sports athletes tended to have lower levels of athletic identity and higher levels of psychological distress. Furthermore, individual sport athletes experience more anxiety and psychological distress than team sports athletes [47]. These studies suggest that in situations such as the COVID-19 pandemic, athletes who participate in individual sports are more likely to experience a lower degree of athletic identity and worse mental health [11,46]. Since the individual sport athletes had lower student-athletes’ identity than the team sport athletes, it would be possible to examine the concerns suggested in previous studies as they apply to Japanese student athletes.

The limitations of this study should be noted. First, the protocol of this study was originally designed to survey the same participants in April 2020 and March 2021 to clarify the relationship between student-athletes’ identity and mental health. However, as of March 2021, the universities that were the target of Study 1 had not resumed athletic activities, and it was not possible to conduct the survey, so Study 2 was conducted on student-athletes who only had resumed athletic activities and were at the same level as Study 1. Originally, it was planned to compare the same participants to examine the two different time phases. However, due to the prolonged COVID-19 pandemic and repeated university shutdowns, the study became different from the original design. In this study, we conducted surveys at one-year intervals to clarify the relationship between athletic identity, mental health, and the impact of social support received from teammates. We hope to conduct future studies with the same participants to gather additional data after the end of the COVID-19 pandemic to gain more insight on this matter. Secondly, the findings of this study were based only on student-athletes earning scholarships. In addition, the study was limited to only male student-athletes as all the teams that consented to the web survey were male. There are approximately 175,000 student-athletes in Japan [48], and the results of this study do not cover the findings of student-athletes of all competitive levels. Future studies should look at a larger sample size, in cooperation with UNIVAS, that involves both male and female student-athletes.

## 5. Conclusions

This study examined the relationships among identity and mental health of Japanese student-athletes under the COVID-19 pandemic. We also examined the relationship with social support received from teammates with the above factors. During Study 1, the first COVID-19 pandemic occurred in Japan. In April 2020, we surveyed social identity and mental health in student-athletes and found a negative correlation between student-athletes’ identity, depression, and sports helplessness. In Study 2 in March 2021, we found a negative correlation between student-athletes’ identity and sports helplessness. In addition, a negative correlation was found between social supports from teammates, depression, and sports helplessness.

## Figures and Tables

**Table 1:** Means (SD) and post hoc analysis for subscales between college years.

Variable	First-Year (*n* = 105)		Second-Year (*n* = 109)		Third-Year (*n* = 83)		Fourth-Year (*n* = 105)		Post Hoc
	Mean	SD	Mean	SD	Mean	SD	Mean	SD	
Student-athlete’s identity	49.83	9.72	46.61	10.69	48.43	10.14	48.16	10.47	n.s.
Depression	7.16	3.14	6.67	3.49	7.33	3.79	7.30	3.61	n.s.
Sports helplessness	6.73	3.30	6.79	3.41	7.47	3.53	8.43	3.78	1st, 2nd < 4th **

Note: ** *p* < 0.01.

**Table 2:** Means (SD) and post hoc analysis for subscales between university year.

Variable	First-Year (*n* = 51)		Second-Year (*n* = 41)		Third-Year (*n* = 44)		Post Hoc
	Mean	SD	Mean	SD	Mean	SD	
Student-athletes’ identity	51.53	9.00	50.68	9.77	50.84	9.23	n.s.
Depression	8.57	3.59	8.56	3.42	7.66	4.32	n.s.
Sports helplessness	7.49	3.31	7.95	3.33	6.77	3.48	n.s.

**Table 3:** Correlation coefficients between student-athlete’s identity with depression and sports helplessness.

Factors/ College Years	1 (AI)				2 (DP)				3 (SH)			
	1st	2nd	3rd	4th	1st	2nd	3rd	4th	1st	2nd	3rd	4th
1 Student-athletes’ identity (AI)	-	-	-	-	−0.01	−0.13	−0.16	−0.16	−0.14	−0.24 *	−0.26 **	−0.30 **
2 Depression (DP)	−0.01	−0.13	−0.16 *	−0.16 *	-	-	-	-	0.52 ***	0.60 ***	0.32 **	0.33 **
3 Sports helplessness (SH)	−0.14	−0.24 *	−0.26 **	−0.30 **	0.52 ***	0.60 ***	0.32 **	0.33 **	-	-	-	-

Note: * *p* < 0.05, ** *p* < 0.01, *** *p* < 0.001, 1st = first-year, 2nd = second-year, 3rd = third-year, 4th = fourth-year.

**Table 4:** Correlation coefficients between student-athletes’ identity, depression, sports helplessness, and social supports from teammates.

Factors/ College Years	1 (AI)			2 (DP)			3 (SH)			4 (SS)		
	1st	2nd	3rd	1st	2nd	3rd	1st	2nd	3rd	1st	2nd	3rd
1 Student-athletes’ identity (AI)	-	-	-	−0.16	−0.19	−0.20	−0.33 *	−0.32 *	−0.47 **	0.37 **	0.42 **	0.56 ***
2 Depression (DP)	−0.16	−0.19	−0.20	-	-	-	0.63 ***	0.66 ***	0.80 ***	−0.43 **	−0.02	−0.14
3 Sports helplessness (SH)	−0.33 *	−0.32 *	−0.47 **	0.63 ***	0.66 ***	0.80 ***	-	-	-	−0.33 *	−0.12	−0.41 **
4 Social supports (SS)	0.37 **	0.41 **	0.56 ***	−0.43 **	−0.02	−0.14	−0.33 *	−0.12	−0.41 **	-	-	-

Note: * *p* < 0.05, ** *p* < 0.01, *** *p* < 0.001, 1st = first-year, 2nd = second-year, 3rd = third-year.

**Table 5:** Results of comparison in student-athletes’ identity and mental health status between the first and second time of the COVID-19 emergency declaration.

Factors	Time of Survey				*t*-Test Results for Time		Effect Size *d*
	1st (April 2020) (*n* = 402)		2nd (March 2021) (*n* = 135)				
	Mean	SD	Mean	SD	*t*	*p*	
1 Student-athletes’ identity (AI)	48.26	10.76	52.55	9.83	4.09	0.000	0.41
2 Depression (DP)	7.48	3.62	8.57	3.85	2.98	0.003	0.30
3 Sports helplessness (SH)	7.26	3.52	7.39	3.39	0.38	0.695	0.16

## Data Availability

Not applicable.
